# Huntington procedure for the treatment of tibial nonunion in a 17-years old male: A case report

**DOI:** 10.1016/j.ijscr.2023.109084

**Published:** 2023-11-20

**Authors:** Nicolò Rossi, Fabio Sciancalepore, Primo Andrea Daolio, Fabio Verdoni, Laura Mangiavini

**Affiliations:** a Residency Program in Orthopaedics and Traumatology, University of Milan, Milano, Italy; b ASST G. Pini-CTO, UOC Ortopedia Oncologica, Milano, Italy; c IRCCS Ospedale Galeazzi Sant'Ambrogio, Milano, Italy; d Department of Biomedical Sciences for Health, University of Milan, Milan, Italy

**Keywords:** Tibial nonunion, Bone loss, Pseudoarthrosis, Huntington procedure, Vascularized fibular autograft

## Abstract

**Introduction:**

Bone is considered a tissue with good healing properties, and many bone defects can heal spontaneously under appropriate conditions. Extreme bone loss can hinder remodeling and regenerative processes, leading to bone nonunion. This condition negatively impacts the patient's quality of life with a severe socioeconomic burden. Many treatment options have been proposed, but none can be defined as a gold standard, mainly due to the variety of clinical presentation, bone loss, and quality.

**Presentation of case:**

We present a 15-year-old case of tibial nonunion following multiple traumas. The patient was treated non-surgically at the beginning, but the external fixator positioning was required due to a delay in the healing process. Following further trauma, the patient showed progressive anterolateral angulation, severe lateral procurvation, and a progressive worsening of the pseudoarthrosis. The severe bone loss and poor quality of the bone surrounding the defect required a special technique called Huntington procedure that consists in a vascularized bone autograft from the ipsilateral fibula to achieve mechanical and biological healing of the pseudoarthrosis. The patient recovered well and returned to full weight bearing without a mobility aid.

**Discussion:**

We report this case of complex tibial nonunion and malalignment, developed after subsequent traumas. Due to the multiple complications, and the poor biology a Huntington procedure was required to provide mechanical stability and a biological boost to the bone defect.

**Conclusion:**

This case report shows a complicated case requiring several surgeries and treatment options and confirms the potential benefit of the Huntington procedure for treating a tibial severe bone loss.

## Highlights

• Bone nonunions represent a clinical and surgical challenge.
• Treatment has many options available, but results are most of the time poor.
• When bone nonunion is present mechanical stability has to be surgically addressed.
• Biological augmentation can help to boost the healing process and improve outcomes.
• Vascularized autograft can provide mechanical stability and biological healing.

## 1 Introduction

Tibial nonunion with massive bone is a challenge for the orthopedic community due to the poor outcomes and the relevant impact on the patient's quality of life [1]. Despite the bone tissue's unique intrinsic healing capacity, pseudoarthrosis has a limited ability for spontaneous repair. This poor healing potential can lead to prolonged hospitalization. It is estimated that the number of new fractures is around 150 million per year, creating a significant socioeconomic burden [2].

For these reasons, the deformities caused by pseudoarthrosis of the tibia must be solved as much as possible. Nonetheless, a gold standard for the treatment of these lesions has yet to be defined [3]. Segmental allograft vascularized, non-vascularized autograft, induced membrane technique, and Huntington procedure are some options available. The bone-transport technique, introduced by Ilizarov, with distraction osteogenesis has represented a valid option [4]. This technique also is not exempt from complications and downsides.

Recently, the importance of restoring the biological microenvironment has gained increasing popularity [5]. The use of a free vascularized bone transfer has been suggested as the leading option to boost the osteointegration process even if challenging due to the highly technical microsurgical skills [6]. Moreover, when a complex tibial nonunion with extensive bone loss is present, the Huntington procedure has been proposed [7,8]. It provides a large graft of the ipsilateral fibula raised on a pedicle of the peroneal artery. This technique, also called fibula pro tibia was first used successfully by Huntington in 1903. In this procedure, the fibula is transferred to the tibia as a pedicle graft and fixed with screw-plate fixation [7]. Due to retained blood supply to one end of the transplant, the graft quickly gets bigger upon weight-bearing. However, the leg-length discrepancy following this treatment is still a concern. To overcome this issue, the Ilizarov distraction-compression osteogenesis technique could be associated with the Huntington procedure [7,8].

We report a 15-year-old case of tibial pseudoarthrosis due to multiple fractures. Our case was treated primarily using an external fixator and then with the Huntington procedure.

## 2 Presentation of case

**Fig. 1:**
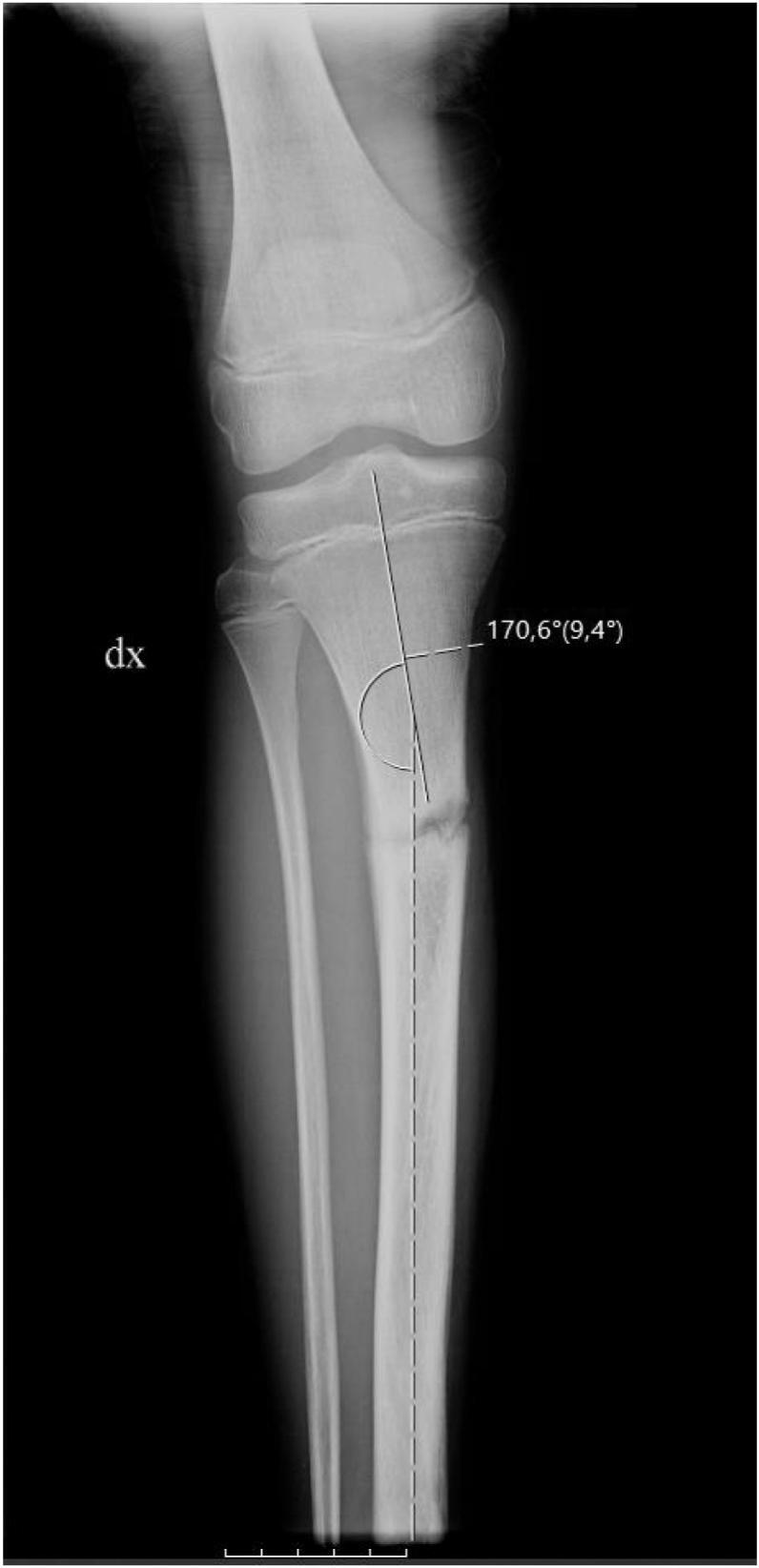
Local X-ray of the right leg 1 year after trauma.

**Fig. 2:**
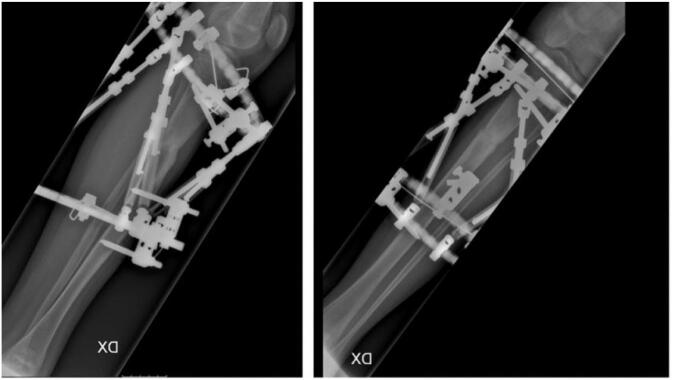
Ilizarov ring fixator application with good bone union.

This case follows 2020 SCARE guidelines for reporting cases in surgery [9]. A 15-year-old male was transferred to our hospital and diagnosed with pseudoarthrosis of the right tibia. He reported a history of right lower limb pain and difficulty with weight bearing of the right lower extremity after a trauma occurred during sports activity (soccer). The x-rays showed a lower 2/3 displaced fracture of the tibia, and he was treated in a local hospital non-surgically with cast immobilization for seven weeks. Unfortunately, the patient continued experiencing pain and weight-bearing difficulties after cast removal. Almost one year after, the patient was admitted to the pediatric unit of the IRCCS Ospedale Galeazzi-Sant'Ambrogio, Milan. Physical examination revealed edema localized to the right distal tibia. Local and plain weight-bearing X-ray and a Ct scan demonstrated an initial anterolateral tibial angulation (Valgus angle: 10°) and procurvation secondary to a hypertrophic nonunion (Fig. 1). Orthopedic consultation recommended management with the Ilizarov ring fixator application that was kept for five months with good fracture healing (Fig. 2). The patient recovered well and returned to full weight-bearing at three months postoperatively.

**Fig. 3:**
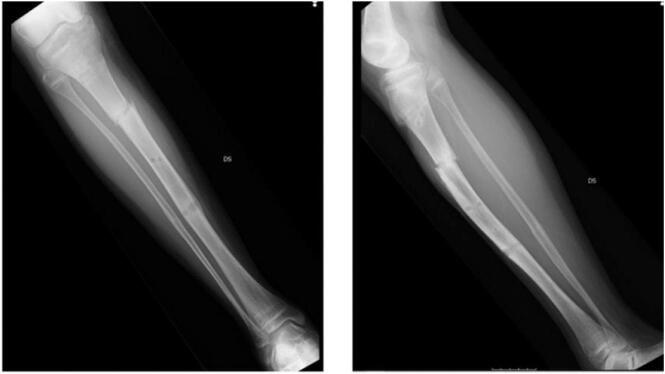
Refracture of the tibia.

Six months after the Ilizarov ring fixator removal, the patient refractured the tibia (Fig. 3) following another sportive trauma and positioning of the Ilizarov ring fixator was required to treat the fracture. During the postoperative follow-up, the patient experienced a Kirschner wire tract infection that was treated with antibiotic therapy, Kirschner wire removal and resolved spontaneously. Nevertheless, a progressive anterolateral tibial angulation and difficulties in bone healing with severe bone loss was seen.

**Fig. 4:**
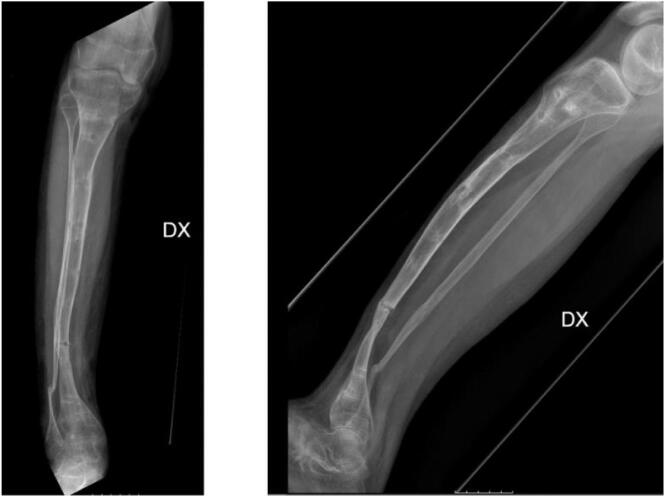
Progressive worsening of the pseudoarthrosis with a thinning of the distal fibula.

Fifteen months after, the external fixator was removed, and a plaster cast was applied from above the knee to the leg to avoid re-fractures for 2 months. During the follow-up care, the patient experienced a new pseudoarthrosis in the distal aspect of the tibia located at the external fixator fish hole with progressive anterolateral angulation (valgus angle 20°), severe procurvation (30°), and a progressive worsening of the pseudoarthrosis with a thinning of the distal fibula (Fig. 4). An MRI evaluation showed swelling of the spongy bone without clinical or radiographic evidence of ongoing infection. The patient underwent bone densitometry test, the genetic analysis, and the blood exams, including anti-cardiolipin antibodies, p-anca, c-anca, anti-Ena, Lupus anticoagulant, C3, C4, and anti-DNA. All laboratory values were within normal limits.

**Fig. 5:**
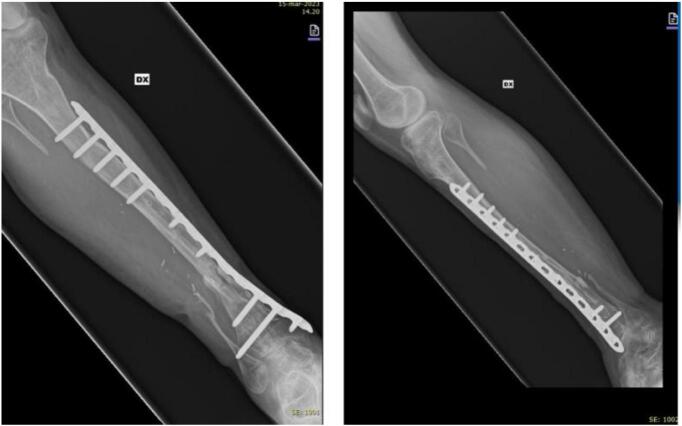
Radiographic outcome of the Huntington procedure.

The patient was then admitted to the “Gaetano Pini Institute”, Milan for an intercalary resection of the tibia segment affected by pseudoarthrosis and reconstruction with the Huntington technique. For surgical exposure, a standard lateral approach was performed. Intraoperative cultures were obtained to confirm absence of ongoing infection. The pseudarthrosis was then debrided and the tibial bone adjacent to the defect was decorticated. A proximal osteotomy at 2 cm below the neck of fibula was performed. Next the flap was raised with standard technique of free micro-vascular flap. The fibula was then osteotomized at both ends with preservation of the peroneal vessels and was shifted in the tibial defect and placed in intramedullary space and fixed with plate and screws (Fig. 5).

The patient recovered well in the postoperative time and was discharged from the hospital five days after. Shortly thereafter, the patient experienced a wound complication with a superficial infection of the wound carried by Staphilococcus Aureus treated with antibiotic therapy and no further complications. The patient recovered well without any progression of the disease.

Currently, one year from the latest surgery, he participates in all activities without pain. At the most recent examination, radiographs demonstrated maintenance of surgical correction and signs of bone healing. He could walk in full weight bearing without any mobility aid with a 10 cm leg-length discrepancy. Now, the patient uses an orthotic treatment to overcome the leg-length discrepancy.

## 3 Discussion

Tibial nonunion represents a surgical challenge due to the complicated treatment issues. The correct timing for diagnosis and treatment is crucial [10]. Surgical options for nonunion bone treatment may include plate osteosynthesis, intramedullary nailing, autograft or allograft augmentation, and bone stimulators. Distraction osteosynthesis through the nonunion site may be especially useful in multiplanar correction cases with low recurrence rates [11].

In this case, a deformity resulting from trauma created a diaphyseal nonunion and a varus malignment. Because there were two deformities at separate levels, resolving the deformity through a single site would have produced significant residual deformity at one of the two levels. The Ilizarov method of distraction osteogenesis allows for the resolution of the stiff hypertrophic nonunion, correction of the varus deformity, and slight lengthening at the diaphyseal nonunion site allowing full weight-bearing. The 6-axis system permits the correction of all axes of deformity simultaneously with a minimally invasive approach [12].

Unfortunately, the patient experienced a further fracture at the same site, impairing bone healing. The new trauma caused a progressive worsening of the tibial nonunion. The extensive bone loss could be managed with various methods [3,6,8].

The most common methods of treating a segmental bone defect are either using a vascularized fibular autograft or by bone transport using the Ilizarov technique [7]. In our case, bone transport could not be proposed due to the impaired bone quality surrounding the tibial nonunion and we proposed a reconstruction with ipsilateral fibula vascularized graft and screw fixation.

Fibula has been used extensively in the pediatric population [13]. Traditionally, the fibula can be used for fibular transposition reverse-pedicle myosseous flaps, but most importantly, fibula-free flaps. The anatomic regions where a free fibula flap can be used for lower extremity reconstruction are from the knee to the forefoot due to its triangular shape capable of withstanding high compressive forces [14]. We decided to treat our patient with the Huntington procedure to provide a viable living bone that increased the osteointegration of the reconstructed bone.

Leg length discrepancy is a common complications when dealing with a critical-size bone defect [15]. If left untreated, this condition can cause long-term complications. Although Vogt et al. [15], in their review, underline the importance of treating the discrepancy when above 2 cm, they also state that it is challenging to standardize the therapy because each patient must be evaluated based on an individual basis, balancing the risks and benefits for the patient. The only presence of a leg length discrepancy does not automatically constitute an indication for treatment. Our treatment option for our patient resulted in a 10 cm leg-length discrepancy treated conservatively with a 4 cm insole. Despite the burden that this discrepancy can create for the patient, the excellent bone healing reached with this technique, the possibility for the patient to achieve full weight bearing without any pain in his daily activities, represents a good outcome. Nevertheless, a question of any subsequent procedure of leg lengthening is still open and will need further discussion due to the skeletal maturity. To date, the patient refused any further surgical procedure at the last follow up.

## 4 Conclusion

We report a surgical case of tibial nonunion treated with Huntington procedure. Regarding tibial nonunion nonresponsive to the Ilizarov distraction-compression osteogenesis technique and other conventional surgical approaches, it is desirable to use the Huntington procedure to provide mechanical and biological healing, specifically in young patients.

## Consent

Written informed consent was obtained from the patient for publication of this case report and accompanying images. A copy of the written consent is available for review by the Editor-in-Chief of this journal on request.

## Funding

This research did not receive any specific grant from funding agencies in the public, commercial, or not-for-profit sectors.

## Ethical approval

No need for an approval from an ethics committee for such a type of article (case report).

## Author contribution

NR drafted the manuscript. NR and LM participated in treating the patients and revised the manuscript. NR, FS, PAD, and FV participated in the surgery and postoperative management. NR and LM were responsible for this paper. All the authors read and approved the final manuscript.

## Guarantor

Dott. Nicolò Rossi.

## Research registration number

None.

## Conflict of interest statement

None.

## References

[1] Van Le D., Van Nguyen L. (2021). Fibula lengthening then centralization for the treatment of pseudoarthrosis at the middle third of tibia with large leg-length discrepancy - a case report. Int. J. Surg. Case Rep..

[2] Greenwald A.S., Boden S.D., Goldberg V.M., Khan Y., Laurencin C.T., Rosier R.N. (2001). Bone-graft substitutes: facts, fictions, and applications. J. Bone Joint Surg. Am..

[3] Enneking W.F., Morris J.L. (1972). Human autologous cortical bone transplants. Clin. Orthop. Relat. Res..

[4] Ilizarov G.A., Ledyaev V.I. (1969). The replacement of long tubular bone defects by lengthening distraction osteotomy of one of the fragments. Clin. Orthop. Relat. Res..

[5] Rossi N., Hadad H., Bejar-Chapa M. (2023). Bone marrow stem cells with tissue-engineered scaffolds for large bone segmental defects. A systematic review. Tissue Eng. Part B Rev..

[6] Feltri P., Solaro L., Di Martino A., Candrian C., Errani C., Filardo G. (2022). Union, complication, reintervention and failure rates of surgical techniques for large diaphyseal defects: a systematic review and meta-analysis. Sci. Rep..

[7] Catagni M.A., Camagni M., Combi A., Ottaviani G. (2006). Medial fibula transport with the Ilizarov frame to treat massive tibial bone loss. Clin. Orthop. Relat. Res..

[8] Khan A.Q., Siddiqui Y.S., Julfiqar, Abbas M., Sabir A.B. (2021). Role of Huntington procedure as a limb salvage surgery for complex gap nonunion of tibia in children. J. Clin. Orthop. Trauma.

[9] Agha R.A., Franchi T., Sohrabi C., Mathew G., Kerwan A. (2020). The SCARE 2020 guideline: updating consensus Surgical CAse REport (SCARE) Guidelines. Int. J. Surg..

[10] Miraj F., Aprilya D. (2020). Diagnostic and treatment challenge in adult presentation of congenital pseudoarthrosis of the tibia: a case report. Ann. Med. Surg. (Lond.).

[11] Koren L., Keren Y., Eidelman M. (2016). Multiplanar deformities correction using Taylor spatial frame in skeletally immature patients. Open Orthop. J..

[12] Abuomira I.E., Sala F., Elbatrawy Y., Lovisetti G., Alati S., Capitani D. (2016). Distraction osteogenesis for tibial nonunion with bone loss using combined Ilizarov and Taylor spatial frames versus a conventional circular frame. Strateg. Trauma Limb. Reconstr..

[13] Reichert J.C., Epari D.R., Wullschleger M.E. (2012). Bone tissue engineering. Reconstruction of critical sized segmental bone defects in the ovine tibia. ORTHOPADE.

[14] Bibbo C. (2021). The free fibula flap for lower extremity reconstruction. Clin. Podiatr. Med. Surg..

[15] Vogt B., Gosheger G., Wirth T., Horn J., Rödl R. (2020). Leg length discrepancy- treatment indications and strategies. Dtsch. Arztebl. Int..

